# On the Preservation of the Teeth

**Published:** 1852-01

**Authors:** H. L. Jacob

**Affiliations:** England.


### ARTICLE III.

*On the Preservation of the Teeth.* By H. L. JACOB, Esq., M. R. C. S., &c., England.

IN the course of my practice I have continually had occasion to deplore the ravages which disease has been permitted to make in the mouths of even young persons, especially young women; and have remarked, that although in some cases this may be traced to carelessness or indifference, it proceeds, in the majority of instances, from sheer ignorance of the nature and extent of the resources of dental surgery, and of the circumstances under which professional assistance is most required and most successfully employed. It is with the hope of diminishing, in some degree, this unfortunate ignorance, and of making it clear to persons of all classes, that it is in their power to save themselves a vast amount of pain, distress, and inconvenience, by a little timely attention, that I am induced to bring these pages before the public.

The evils attendant on the loss of the teeth, must be pretty obvious to all; an unsightly appearance, an aspect of premature old age, from the falling in of the lips and cheeks, lisping and other imperfections in the articulation of the voice, injury to the health, resulting from the imperfect mastication of the food, and the continual presence of pain and irritation about the mouth; all these, as well as the suffering, inconvenience, and personal discomfort which disorders of the teeth occasion, are so palpable and evident, that it might, perhaps, be deemed superflous in me to inculcate the prudence of taking pains to avoid these evils; but I would insist on a point which is, perhaps, not quite so obvious, namely, that it is not only very foolish, but also very *reprehensible*, to neglect the state of our mouths. It is a positive duty we owe to society to avoid, as far as possible, giving offence to the senses and feelings of others; and what can be more disgusting than a display of swollen ulcerated gums, and a parcel of black ragged remains of teeth in various stages of decay; or a foul incrustation of the accumulated secretions of years, enveloping the teeth and gums in one general mass of filth, and tainting the air with the most noisome exhalations? I maintain that persons have no right to shock our senses by these offensive exhibitions, and thus make themselves nuisances to society, when it is in their power to avoid it; and there are very few, in this part of the world at all events, who do not possess that power. It is useless to say the poor creatures cannot help being afflicted in this manner; in all cases this state of things admits of improvement, and in nearly all it might have been prevented altogether. Many, as I stated at the commencement, suffer in this way through ignorance; it is high time, then, that they should be enlightened on this point; as for those who wilfully allow their mouths to get into this filthy condition, if they are not to be acted on by a sense of delicacy and propriety, they may, perhaps, be deterred by a sense of *shame*, from incurring the charge of uncleanliness, a fault which society is very quick to perceive, and very slow to forgive. In individuals of the fair sex especially, such neglect is unpardonable; it renders repulsive an otherwise pleasing and attractive person, while there is no feature more agreeable and prepossessing than a good clean set of teeth; in fact, to borrow the words of a French author, "with bad teeth no woman was ever beautiful; with good teeth no woman was ever plain."

I shall now proceed to offer a few words of advice respecting some of the commoner and more important disorders of the teeth, with the means to be adopted for relieving or preventing them, and endeavor to correct sundry erroneous notions which I have observed to prevail very generally on these subjects; not with a view of giving the public any instruction in the art of dentistry; an attempt to do this in a small pamphlet, even were it desirable, would be useless and ridiculous ; but simply that people may have some idea of what dental surgery can or cannot do, and no longer, on the one hand, deprive themselves of the benefits which science has placed at their command through ignorance of their existence, nor, on the other, feel so much confidence in the resources of the dental surgeon, as to expect something like a miracle to be performed on their behalf.

First, then, for that commonest of all disorders of the teeth, CARIES OR DECAY. This, which is so well known as to need no description, is by far the most frequent cause of the destruction and loss of the teeth, and almost the only source of genuine *tooth-ache*, the tortures of which most individuals have experienced at some period of their lives. Now, of the many thousands of teeth that are annually lost from this cause, there is scarcely one that might not have been preserved by proper care and attention ; and I am convinced that if this fact were more generally known, people would not be content, as so many now are, to let their teeth go on decaying year after year, and subjecting them to continual pain and inconvenience. I would urge more particularly the importance of having the teeth attended to in early life, that any defects or irregularities in their growth may be at once detected and rectified before any permanent mischief has been produced ; it is surely inconsistent with common humanity to neglect such simple means of preventing so much subsequent distress and suffering to the rising generation ; and I would suggest to all parents the propriety of having their children's mouths examined by some respectable practitioner in dental surgery, or, at all events, by their medical attendant, as soon as the teeth of the second set begin to make their appearance. If, on examination, it is found that every thing is going on right, they will have the satisfaction of knowing that such is the case ; if, on the contrary, there should be some defect which requires to be remedied, they will have the happiness of reflecting that their child has not suffered through their neglect. This examination should be repeated two or three times a year, in some cases oftener, during the whole period of the shedding of the teeth. One important reason for this attention, is the fact that the teeth of the second set being in general much larger than those of the first, it frequently happens that the jaws do not undergo an increase of dimensions sufficient for the increased space required by the teeth; these are consequently crowded and pressed together with great force, which bruises and crushes their structure; and this, not to mention the unsightly appearance and other inconveniences often produced by the teeth overlapping and growing out of their proper place, is *one of the commonest and most fertile sources of* DECAY; it is to this point, therefore, that I wish more particularly to direct attention, as especially connected with the subject now under consideration. I have seen numerous instances of persons who have lost nearly all their teeth while still young from this cause; now surely it would have been very much better to have prevented this misfortune—and it might in all cases have been prevented with very little trouble and at a trifling cost. It is not only during childhood, however, that means should be taken for preventing the destruction of the teeth by decay; teeth may decay at any period of life, owing to certain original defects in their structure, to uncleanliness, to an unhealthy state of the secretions of the mouth, or of the system at large, &c., as well as to the injury inflicted on them by being over-crowded; and in all cases after decay has commenced it may be arrested and completely remedied, if attended to before it has penetrated deeply into the interior of the tooth; as it generally happens, however, that the individual himself does not become aware of the existence of the decay until it has made considerable progress, frequently, indeed, not until it is too late to remedy it, and as it is of importance that it should be taken in hand at an early stage, it will be prudent for all who wish to preserve their teeth and escape tooth-ache, to have their mouths examined once or twice a year by some dental surgeon, in whose skill and integrity they can confide, in order that any decay which may exist may be discovered and remedied at once. It is a common thing for persons to say, "there is nothing the matter with my teeth, I never have any pain in them, when I do I will go and have something done."

Now, if they wait until they begin to suffer from tooth-ache before they apply for professional assistance, they will, in nine cases out of ten, apply too late for the preservation of the tooth, or, at all events, too late for any means to be employed for that purpose with any *certainty of success* ; and this fact cannot be too much insisted on. Every body is ready to admit the truth of the maxim, that *prevention is better than cure*, yet, strange to say, some persons seem to prefer to suffer continually from tooth-ache, and to lose their teeth one after another, after putting themselves to much trouble and expense in fruitless attempts to *cure* the pain, rather than have recourse to means which would have *prevented it altogether*, and saved their teeth into the bargain; people inquire eagerly for nostrums to *cure* the tooth-ache, but few appear to be at all solicitous about *preventing* it.

I have said that the progress of decay in a tooth may be arrested, and the injury effectually remedied, if it be taken in hand at the proper time; this is effected by the operation of STOPPING, as it is termed, which consists in removing the whole of that portion of the tooth which is softened by the decay, and filling up the cavity completely with some hard incorrodible substance, so as to entirely exclude all air and moisture ; when this has been properly and skillfully done, the tooth is to all intents and purposes as good as if it had not been decayed at all. The material best adapted for this purpose, generally speaking, is gold, prepared in sheets or leaves ; a piece of this gold foil, of the proper size, is folded or rolled up into a convenient form, and pressed gradually bit by bit into the cavity, until it is completely filled, the gold being packed and pressed together in one solid mass, moulded to the shape of the hole ; silver foil and pure tin foil are sometimes used, but gold is to be preferred. It often happens, however, that owing to certain circumstances, such as the situation of the decayed part, or the great loss of substance which the tooth has undergone, &c., this mode of stopping cannot be employed ; a sort of metallic paste or amalgam is then used, which is introduced into the cavity in a soft state, and in a short time *sets* or hardens in the tooth ; this is a very useful kind of stopping, as it can be moulded to any shape, and thus may be used when a very large portion of the tooth is decayed and broken away; there are various kinds of these metallic pastes, some more useful than others; they are all liable to this objection, that they are apt to become more or less discolored after a time, and to give the tooth a dark disagreeable appearance; where the tooth is out of sight, however, this is of little or no consequence. Some of these substances, under the names of "mineral cement," "succedaneum," "enamel," &c., are advertised extensively by certain ignorant or unprincipled individuals, and sold to the public to enable persons to stop their own teeth and *cure tooth-ache;* and much mischief is thus produced. Some of these substances would doubtless answer very well if properly used; but whatever be the material employed for stopping a tooth, it is absolutely necessary for the success of the operation, *that all the decayed part should be completely removed first;* in many cases it is impossible for any person to do this for himself, and in all cases proper instruments and apparatus are required for the purpose, together with a certain amount of skill and practice in their use; when the tooth is not properly prepared in this way, the decay generally goes on as bad as ever, in some cases even more rapidly than before; moreover, a considerable degree of care and nicety is generally required in the introduction of the stopping; various inconveniences result from its being done in a clumsy manner, and thus, not only is the tooth not preserved, but positive mischief is often occasioned besides: this is not all however; in many instances the cement or "succedaneum" is not inserted into the tooth until it has begun to ache; in this case the pain is generally increased to a tenfold degree, and the patient is obliged to have the tooth extracted to escape from the agony; in some cases the already inflamed nerve is so irritated by the stopping, that matter forms inside the tooth, abscesses arise in the face, other teeth besides the one originally affected are lost, and even portions of the jaw destroyed. In addition to these direct evils, other indirect ones are produced; persons being led to believe that they can stop their teeth and cure tooth-ache so nicely themselves, are lulled into a false security and prevented from having recourse to the proper means for preserving their teeth; and when they find that they have been deceived by the delusive advertisements, sometimes conceive a prejudice against the operation of *stopping*, and take for granted, that because their own ill-advised and imperfect attempts have failed, it is never of any use to have teeth stopped at all; and thus deprive themselves, and perhaps their friends, of one of the greatest benefits that science has conferred on mankind.

A decayed tooth ought to be stopped before the decay has penetrated to the central pulp or *nerve;* when the nerve is exposed, it is too late to have recourse to the operation of stopping with a reasonable prospect of its being permanently successful; it is true that many teeth are stopped under those circumstances, after the nerve has been first destroyed artificially, or has decayed away of itself, and with perfect success, but there is no certainty about it; it not unfrequently happens that the tooth becomes very painful afterwards, and the stopping has to be removed or the tooth extracted; this sometimes occur soon after the operation, but it may be brought on at any time by exposure to cold or some derangement of the general health: sometimes a succession of small abscesses or gum-boils takes place, or the tooth is continually tender and uneasy: this is, of course, very unsatisfactory both to the patient and to the operator; besides the operation of destroying the nerve of the tooth is generally attended with a great deal of pain, and this is another reason why we should take care to have our teeth stopped before the nerve has been exposed, as we shall then avoid this painful process.

As tooth-ache, in almost every instance, arises from the exposure of the nerve by decay, it is evident that when a tooth has begun to ache severely, the chances of being able to preserve it are very much diminished, if not altogether lost. The operation of stopping, when performed at the proper time, is generally attended with little or no uneasiness; but it sometimes happens that a tooth will be somewhat tender and sensitive, when the decay has made as yet but little progress; and the touch of the instrument used to cut away the decayed part, occasions then more or less pain; this is very different, however, from the acute pain that is felt when the nerve is touched; and when the tooth has been restored to a healthy condition by the unsound part being removed, and the cavity properly filled, this tenderness gradually subsides and passes away altogether. When, in consequence of the extreme tenderness of a tooth, it cannot be stopped with any hard metallic substance, some softer material, such as gum mastic, or gutta percha, may occasionally be employed with advantage, as a temporary stopping. I have alluded to *want of cleanliness* as one of the circumstances giving rise to decay of the teeth; when *tartar* is allowed to collect around the teeth, it serves as a reservoir for unhealthy secretions, sour and putrid remains of food, &c., which corrode away the teeth slowly but surely; the decay sometimes goes on so insidiously beneath the coating of tartar, that the teeth are nearly cut through by it before the person is aware that there is anything the matter; there are other evils resulting from neglecting to clean the teeth, which I shall mention presently, but this alone ought to be sufficient inducement for every one to keep his teeth as clean as possible. Some persons, in consequence of decay in their teeth having been brought to light by the tartar being removed, have imagined that the teeth decayed *in consequence* of the removal of this substance, and say that they never knew what it was to have decayed teeth until they had them cleaned; very likely they did not *know* that they had decayed teeth, but it is no less true, that the mischief had been going on for some time previously. A man, who on looking at himself in a glass, discovers that he has a dirty face, might just as reasonably maintain, that his looking in the glass was the *cause* of his face being dirty. It happens sometimes that the teeth decay away very rapidly, in consequence of an unhealthy state of the constitution, the secretions of the mouth being acid and unwholesome, and the teeth at the same time in a weak state, and less able to resist the corrosive action; in this case, we must not confine our attention to the teeth themselves, but must take proper means for restoring the general health. Before quitting the subject of decay of the teeth, I may as well say a few words on TOOTH-ACHE. I have frequently heard persons express a wish that some cure could be found for tooth-ache; well, there are a great many remedies for it; but, setting aside the extraction of the tooth, there is no one plan that will answer equally in all cases; the treatment of this, like that of any other ache or pain, will depend on the circumstances of the case,—an application which, in some instances, acts like a charm, and gives instantaneous relief, will, in others, be perfectly useless, or even aggravate the evil. Sometimes all local applications will be useless, the tooth-ache being kept up by a disordered state of the system, for which the proper remedies must be employed. But if the *cure* of tooth-ache be troublesome and uncertain, we have the satisfaction of knowing that its *prevention* is by no means so. By taking care not to allow the teeth to be crowded and pressed together, by keeping them clean, by not using them for improper purposes, by taking plain wholesome food, avoiding an excess of acids or sweets, and by maintaining a healthy condition of the digestive organs, we shall almost always prevent the teeth decaying at all;—but, if decay should take place, we have it in our power to arrest its progress, prevent tooth-ache, and preserve the tooth, by a timely recourse to the operation of STOPPING.

The condition of the mouth properly known as "SCURVY OF THE GUMS," although not so common as the disease we have have just been considering, is yet frequently met with, and occasions much suffering and inconvenience. This complaint varies a good deal in different cases; in fact, there are, in all probability, several different disorders comprised under the popular term "SCURVY." In some forms of it, the gums are soft, spongy, and swollen—in some instances so much so, as nearly to cover the tops of the teeth;—this is generally accompanied by a large deposit of tartar, and, not unfrequently, by the secretion of a foul, acrid, offensive discharge;—this condition may generally be remedied without much difficulty, but if allowed to go on, it occasions the destruction of the teeth, which either decay away, or gradually loosen and fall out. Another variety of this affection consists in the gradual detachment of the gums from the teeth, apparently without the former being diseased—the disorder consisting in the destruction of the *periosteum* or membrane which covers the root of the tooth, and by which it is attached to the gum and to the bony socket—together with the absorption, to a certain extent, of the bony socket itself, so that an instrument may be passed quite round the root of the tooth, between it and the gum, to a considerable depth ;—this separation of the tooth from the surrounding parts, goes on until it drops out altogether, or until the pain and irritation it occasions, obliges the patient to have it removed. The tooth in these cases is very loose, moving freely in all directions, and acts like a thorn in the flesh, keeping up a continual irritation in the jaw, and occasioning excessive pain each time it meets with a sudden jar or shock. This disease sometimes attacks nearly all the teeth at once, sometimes only one or two, and occasionally a tooth is only partially affected by it, one side of the root (or one of the roots, if there are more than one) being still firmly attached, while the other is quite separated from the surrounding parts. It appears to depend, in some instances, on a certain peculiarity of constitution, which is often hereditary,—in others, it is brought on by salivation from mercurial medicines,—by want of cleanliness,—by the use of hot, stimulating food or other injurious substances,—by derangement of the stomach, liver, &c. ; and two or more of these causes may co-operate at once ; but I have observed it to occur most frequently when the teeth had been more or less *crowded* in the jaw, but, owing to their strength and soundness, and the healthy state of the secretions of the mouth, and the constitution generally, had not undergone decay, but had remained perfect :—in these cases, although the teeth themselves escaped, the injurious effects are felt in the jaw ; and persons who had congratulated themselves on having preserved a complete set of teeth up to the middle period of life, have then been astonished and dismayed at finding them loosening and falling out one after another in quick succession. This furnishes an additional reason for taking steps in early life to prevent the crowding of teeth;—by the help of this and other precautions, we may succeed in preventing the disease from coming on, and by the application of appropriate remedies, it may often be checked after it has commenced; but that portion of the root of the tooth which is already separated from its attachments can never reunite itself to the gum; all we can do is to prevent the mischief from extending farther. When only one or two teeth are affected, they may, in some cases, be tied to the adjoining ones, provided these are firm and sound, by ligatures of fine gold or platinum wire:—the teeth are thus kept steady and rendered much more serviceable and comfortable. When the teeth are fixed in this manner, great care must be taken to keep them clean;—if the ligatures are allowed to get foul, the teeth are apt to become corroded by them.

There is another form of this affection, in which the gums and bony sockets of the teeth become gradually absorbed, and shrink away, so as to lay bare the roots of the teeth; but still the part which remains imbedded in the jaw, continues firmly attached, and it is not until the disease has reached its last stage, and the teeth hang only by the tips of their roots, that much inconvenience is experienced. This complaint is not quite so common as the one last treated of, but it appears to result from similar causes, in fact, the two forms of disease are not unfrequently found co-existing in the same mouth, and more or less blended together. Although it occasions less pain and inconvenience than the former one, it is even less under the control of remedies;—we may generally check and retard its progress to a certain extent, but can seldom succeed in stopping it altogether,—and, of course, the loss of substance that has actually taken place, can never be restored. Doubtless much more might be done in the way of remedying these affections, if those who suffered from them had patience and resolution enough to persevere in the treatment; but as this generally has to be continued for a considerable time,—and constant care and attention is requisite to prevent a recurrence of the disorder, many persons think that the remedy is worse than the disease, and would rather lose their teeth than take so much trouble, and deprive themselves of certain luxuries in the way of eating and drinking, &c., in order to preserve them.

I have two or three times had occasion to allude to the accumulation in the mouth of the substance called "TARTAR;"—this consists chiefly of various earthy matters deposited from the saliva,—and, in certain unhealthy conditions of the mouth, or of the system generally, it collects round the teeth in large quantities;—but there are few individuals who are not subject to it more or less. I have mentioned one of the evils arising from its presence, namely, decay of the teeth; it also keeps the gums in a continual state of irritation and tenderness, causes them to recede from the teeth, and by insinuating itself deeper and deeper along the roots of the teeth, destroys their connection with the jaw, and causes them to get loose and fall out. Being porous, it serves also as a reservoir for all sorts of fluids, such as the juices of the food, secretions from the gums, &c.—which are continually undergoing putrefaction, and thus it renders the breath offensive, and occasions a foul, unpleasant taste in the mouth; where it is visible, it is equally disgusting to the eye;—for all these reasons, it is incumbent on all who value their own comfort, and have any consideration for the feelings of others, to spare no pains to keep their teeth free from this filthy incrustation. Where the teeth have been from childhood regularly and properly cleaned, inside and out, the tartar is prevented from ever accumulating, but when it has accumulated in consequence of neglect, the ordinary mode of cleaning with a brush will not remove the evil;—the operation of SCALING must then be performed,—and when the teeth have been scraped or *scaled* quite free from the tartar, they may then be kept clean for the future, by the use of the brush and appropriate tooth-powder. I believe some persons object to the operation of scaling, in consequence of some very curious fancies,—such as that it is very painful,—that it injures the enamel of the teeth, causes them to decay, makes them loose, &c.; it is difficult to imagine how such erroneous ideas could have arisen; the operation is attended with little or no pain, and inflicts not the smallest injury on the teeth, but, on the contrary, preserves them from decay and destruction; I have already made some remarks on this subject as far as relates to *decay*; with respect to the loosening the teeth, this is produced by the *presence* of the tartar, not by its *removal*, which prevents the further progress of the mischief, and allows the gums a chance of returning to a firmer and more healthy condition. I have known some individuals excuse themselves from even the use of the tooth-brush, on the pretext that it injures the teeth to clean them; this opinion has perhaps arisen in consequence of some injurious substance being employed for that purpose, as I shall presently explain; the cleaning of the teeth, when done in a proper manner, is one of the most effectual means of preserving them. All persons ought to clean their teeth, at least once a day, some require to do so oftener, even after every meal, as well as on rising in the morning: in some few individuals, the mouth is in such a healthy state, that water alone is sufficient for the purpose, but most people require the aid of tooth-powder; this must be suited to the nature of each case—that which would be perfectly effectual in some cases, would be of little or no avail in others; and now that I am speaking of tooth-powder, I may as well caution the public against the indiscriminate and excessive use of certain substances which are much employed for this purpose. Camphor is a very favorite ingredient in tooth-powders, and it is very fragrant and refreshing, but I have known it in many cases give rise to very serious mischief, the teeth becoming deeply notched on their external surface, close to the gum, just as though they had been cut away with a three cornered file; sometimes there is not much loss of substance, but the teeth become very tender and sensitive to heat and cold, and at length decay away close to the gum. Charcoal also, which is another substance much used as tooth-powder, is apt to cut the teeth in the same way.

With regard to the various "Odontos" and "Dentifrices" which are puffed and advertised into notoriety, and which are retailed by parties who are utterly ignorant of their nature and composition, but who nevertheless take all the pains in their power to recommend them to the public, I can only say, that without knowing precisely what they consist of, no positive opinion could be given as to whether they were likely to be beneficial, harmless or injurious; but I would caution persons against the use of those dentifrices which profess to "*whiten*" the teeth; if they have this effect, they most probably contain some ingredient of an acid nature, which although it would have the effect of "*whitening*" the teeth at first, would render them more liable to become discolored afterwards, and which by habitual use would completely destroy them. The surface of the teeth should be kept as *clean* as possible, but no attempt should be made to alter the color of the teeth themselves; if the discoloration is produced merely by tartar, or other matters deposited on their *surface*, it may and *ought* to be remedied by the removal of the tartar, &c., by mechanical means; but when the *substance* of the teeth is discolored, any endeavor to "whiten" them by acids or other chemical agents, will be not only useless but mischievous. Besides scouring the surface of the teeth from all extraneous matters, the dentifrice should have the effect of checking the formation of the tartar, rendering the gums firm and sound, and keeping the secretions of the mouth in a healthy state. Some persons prefer a dentifrice prepared in the form of a *paste*, but I think that, on the whole, the form of a *powder* is most effectual and convenient. As to what is *the best kind of tooth-powder*, this, as I mentioned before, will depend on the nature and circumstances of each case; there is no one kind that would suit all persons indiscriminately, and people would do well to bear this in mind, and not go and buy tooth-powder in the same way that they would buy a box of plate powder, or a jar of blacking.

I do not intend now to enter minutely into the subject of ARTIFICIAL TEETH; I will merely remark with respect to them, that notwithstanding they have been brought to such a degree of perfection, as to serve almost all the purposes of those with which nature has provided us, yet the process of mastication is always somewhat better performed with good, sound teeth of our own, than with the best artificial substitutes : moreover, artificial teeth cannot be supplied equally well and successfully in all cases ; it will be prudent, therefore, to take all the means in our power to preserve our own teeth in good condition, and not, as I have known to be the case with some persons, be careless about the preservation of these useful and important organs, in the expectation of finding no difficulty in supplying their loss with artificial ones; such over confidence in the resources of the dental art, should be avoided ; but, at the same time, we should bear in mind, that well constructed teeth will enable us to masticate our food very much better than we can do when our own are loose, imperfect, or deficient. I may as well mention, that one serious objection which formerly existed to the use of artificial teeth, namely, the disagreeable idea of wearing teeth which had been taken from the mouths of dead persons, is now completely removed by the introduction of porcelain or *mineral* teeth, which are manufactured of great beauty, and in close imitation of nature ; these have also the advantages of superior cleanliness and durability, as they never corrode or decay in the smallest degree. The improvements that have been made in the art of mechanical dentistry during the last twenty or thirty years are very great ; and as the progress of improvement tends also to facilitate labor and diminish expense, the resources of the dental art are brought more and more within the reach of the public, and the number of those who avail themselves of artificial teeth is continually on the increase.

I should have liked to make a few observations on various other matters connected with the teeth, especially the serious injury to the general health, which frequently arises from a diseased state of the mouth ; entailing on the patient much suffering and distress, in addition to the more immediate effects of the local disorder, such as tooth-ache, swelled face, tic douloureux, &c.—but I have already extended my remarks over a larger space than I at first intended, and I trust I have said sufficient to induce people in general to pay more attention to their teeth, and no longer neglect those means which are requisite for their preservation. Timely care and attention are to be recommended even on the score of *economy ;* many persons after they have lost their teeth through neglect, find themselves obliged to procure artificial ones at a much greater expense than the preservation of their own would have occasioned them ; and those who are suffering from tooth-ache would gladly pay double as much to get it *cured*, as they would have had to pay for its prevention. It may be objected that there are numbers of persons who, although they are fully aware of the evil consequences arising from neglect of the teeth, and would gladly avail themselves of the assistance of the dentist in good time, are prevented from doing so by a fear of being unable to afford the necessary expense. I believe much misapprehension prevails on this point; the operations of dentistry are in general not so costly as many persons are apt to imagine ; the earlier the mischief is attended to, the less will be the expense; and many persons actually pay more for the extraction of their teeth, and for nostrums to cure the tooth-ache, than they would have had to pay for the preservation of their teeth, and the prevention of tooth-ache altogether. However, granting that the operations of dental surgery are somewhat expensive, a considerable reduction would always be made in favor of the working classes, as in the case of medical and surgical attendance generally; moreover, I see no reason why dispensaries for diseases of the teeth should not be established, not exactly as *charities*, but rather as *clubs*, "self-supporting" dispensaries as they are called, where people, by the annual payment of a few shillings, could have their teeth kept in order, and all the requisite operations performed for their preservation ; we have hospitals, infirmaries, and dispensaries, for all other diseases except those of the teeth and gums, and it is high time that this deficiency was remedied. I am confident that institutions of this sort will be established at some future time, and then every body will wonder how it was they were not set on foot sooner. Up to a recent period the assistance of the dental surgeon has been regarded in the light of a *luxury*, to be indulged in only by the wealthy and higher classes ; this opinion is now, by the march of civilization and improvement, fast disappearing, and before the lapse of many years, will, I trust, have almost ceased to exist among us. It will, doubtless, be the same with this as with the other arts of life; that which to our forefathers was a rare and costly luxury, will, to our posterity, be an ordinary and almost necessary comfort, enjoyed by every, or nearly every, class in society.

I shall be rejoiced if I am able in any way to contribute towards hastening this diffusion of the benefits of science, as regards this branch of the healing art, and thus be instrumental in bringing about a much more widely spread prevention or alleviation of some of the numerous sufferings to which mankind is subject.

*Bridgwater, January*, 1851.

